# Locomotive Syndrome Digital Therapeutics Provided via a Smartphone App: Protocol for a Single-Group Trial

**DOI:** 10.2196/70163

**Published:** 2025-04-17

**Authors:** Tatsuru Sonobe, Yoshihiro Matsumoto

**Affiliations:** 1 Department of Orthopaedic Surgery Fukushima Medical University School of Medicine Fukushima Japan

**Keywords:** locomotive syndrome, locomotion training, digital therapeutic, TUG, 25-Geriatric Locomotive Function Scale, BREQ-3, behavioral change, support application

## Abstract

**Background:**

Locomotive syndrome (LS) is a condition in which muscle weakness and reduced motor function due to musculoskeletal disorders cause reduced mobility and physical function. In Japan, musculoskeletal disorders are the most frequent reason for requiring home support or nursing care, and the prevention and amelioration of LS are thus being emphasized. However, it is difficult for older people to make a habit of exercise therapy, which is the mainstay of LS treatment. We investigated whether digital therapy could (1) lead to behavioral change in older people and (2) prevent or improve LS in older people.

**Objective:**

We sought to determine whether digital therapeutics (DTx) are useful for the prevention and amelioration of LS in older people, and we assessed the effects of DTx on the participants’ exercise awareness and motor function.

**Methods:**

We conducted a multicenter, prospective, longitudinal, nonrandomized, single-group study of Japanese adults aged ≥40 years who were eligible for LS checks. Each participant underwent an 8-week locomotion training (LT) intervention, and their subjective and objective motor abilities and motor awareness were objectively assessed at the following time points: baseline (before the start of the DTx), interim (4 weeks after the start of the DTx), and end (8 weeks after the start of the DTx). We evaluated the participants’ objective motor function using the timed up and go (TUG) test, and we compare the results using a 3-way ANOVA with the TUG test at the 3 evaluation time points as the dependent variable. The results of the 25-question Geriatric Locomotive Function Scale, which is a subjective measure of motor function, and the results of the Behavioural Regulation in Exercise Questionnaire 3, which assesses motor awareness, were also evaluated using an ANOVA in the same way as the TUG test. The significance level was set at .05 / 3 = .0167 after Bonferroni correction.

**Results:**

As of April 2025, this study had enrolled 47 participants, and complete data had been gathered from 45 participants for the proposed analysis. Study participation was ongoing as of April 2025.

**Conclusions:**

The study cohort will be used as a basis for further observational and intervention studies. This research could lead to more efficient use of medical resources and a reduction in financial and medical burdens on individuals and the economy, and it could support the prevention and amelioration of LS and the establishment of exercise habits among older people.

**Trial Registration:**

University Hospital Medical Information Network Clinical Trials Registry UMIN000053922; https://center6.umin.ac.jp/cgi-open-bin/ctr_e/ctr_view.cgi?recptno=R000061550

**International Registered Report Identifier (IRRID):**

DERR1-10.2196/70163

## Introduction

### Background and Rationale

The Japanese Orthopaedic Association (JOA) issued its first guidelines regarding locomotive syndrome (LS) in 2007 [1]. Locomotive syndrome refers to a condition in which a musculoskeletal disorder results in muscle weakness and motor weakness, leading to reduced mobility and physical function [1]. The development of LS causes a decline in healthy life expectancy and can lead to individuals becoming bedridden and requiring long-term care [2]. In Japan, motor system disorders are the most frequent reason for requiring assistance at home or nursing care, accounting for 24.8% of all cases [3]. The JOA recommends the early detection of motor function decline by conducting LS checks (LSCs), in part to prevent the future need for assistance or care [4]. The JOA also advocates locomotion training (LT), which involves standing on one leg and squatting, to prevent and ameliorate LS [5].

Guidelines for assessing the risk of LS were developed in 2015 [6]; these defined stage 1 LS as the early stage of motor decline and stage 2 LS as advanced motor decline. In 2020, stage 3 LS was added, defined as a state of progressive decline in mobility and difficulty in social participation [7]. The risk of developing LS is assessed based on three items: (1) a stand-up test, (2) a 2-step test, and (3) the 25-question Geriatric Locomotive Function Scale (GLFS-25) [5].

Japan has one of the world’s most prominent aging populations, and the burden of health care for the oldest segment of Japan’s population on the country’s insurance system continues to increase [8]. It is also necessary to consider how to make the most effective use of limited medical resources. The prevention and amelioration of LS are considered important as countermeasures against motor system disorders. Exercises such as LT are effective for preventing and ameliorating LS [5], but it is difficult for many older adults to start or continue an exercise routine. As one solution, we advocate an approach to the prevention and improvement of LS in older people based on using technological devices to promote behavioral change.

Technology is used in the medical field for electronic medical records, telemedicine, and surgical robots; in addition to this, mobile devices are used for medical procedures and support, which is called mobile health (mHealth) [9]. Digital therapeutics (DTx) are an area of mHealth that is receiving particular attention [10]. A variety of apps have been approved by insurance as DTx, including diabetes and hypertension self-management apps and smoking cessation support apps [11-13]. However, DTx for musculoskeletal diseases have not been approved for insurance coverage. Although some apps have functions such as step counting and exercise management, they are not scientifically supported. We have created a smartphone app for older people who have no exercise habits and whose physical functions have declined. The smartphone app has functions that support the implementation of 2 types of LT, using video and audio, and when participants carry out LT, the implementation status is recorded not only on the target person’s smartphone, but also on the health care provider’s smartphone. The advantage of this system is that health care providers can check the progress of LT remotely, and the patient can feel that they are exercising with the support of health care providers. We are conducting this study to determine whether the use of DTx can lead to behavioral change in older people and prevent or improve LS.

### Objectives

The study’s primary aim is to determine whether the use of a smartphone app (ie, DTx) can prevent or ameliorate LS in older people: the outcomes are exercise awareness as assessed by the Behavioural Regulation in Exercise Questionnaire 3 (BREQ-3) and motor function as assessed by the GLFS-25 and timed up and go (TUG) test. In this study, participants who were unable to perform the stand-up test or 2-step test due to hip or knee pain were excluded from the measurement items. The research questions are as follows: (1) Can older people use DTx to proactively change their exercise habits with the help of their health care providers? (2) Are changes in exercise habits in older people associated with changes in motor awareness and function?

Our main and secondary study hypotheses are that (1) continued LT with DTx will prevent or improve LS in older adults and (2) well-established exercise habits will improve these individuals’ motor awareness and function.

## Methods

### Participants and Study Setting

We plan to recruit a minimum of 50 participants aged ≥40 years who are eligible for LSCs [14] at any of 3 general hospitals (Bange-Kosei General Hospital, Southern Tohoku General Hospital, and Minami-Soma City General Hospital) that perform orthopedic surgery. The target minimum numbers of participants is 25 at Bange-Kosei General Hospital, 15 at Southern Tohoku General Hospital, and 10 at Minami-Soma City General Hospital. The overall recruitment target is based on the minimum sample size necessary to complete the intended analysis, taking omissions into account.

The study’s inclusion criteria are as follows: participants aged ≥40 years who generally have lower-extremity muscle weakness [15] and who have visited 1 of the 3 abovementioned hospitals for an LSC, which are scored as follows: 1 point if the individual cannot put on a sock on one foot; 2 points if they stumble in their house; 3 points if they need a handrail to climb stairs; 4 points if they have difficulty using a vacuum cleaner at home; 5 points if they have difficulty carrying parcels weighing >2 kg; 6 points if they have difficulty walking continuously for >15 minutes; and 7 points if they cannot cross a crosswalk with a green light. Additional inclusion criteria are scoring ≥3 points on the Mini-Cog cognitive functional assessment [16,17], having access to the smartphone app, and being able to provide informed consent for study participation. The exclusion criterion was difficulty using smartphone apps due to cognitive decline or visual or hearing impairment.

### Study Design

This trial is a multicenter, prospective, longitudinal, nonrandomized, single-group study of Japanese adults aged ≥40 years with applicable LSC results. The design includes 3 evaluation time points: baseline (0 weeks, ie, before the initiation of DTx), an interim evaluation (4 weeks after DTx initiation), and a final evaluation (8 weeks after DTx initiation).

An investigation of DTx that was designed to reduce anxiety about recurrence in postoperative breast cancer patients included a baseline assessment before the DTx intervention, an interim assessment after 4 weeks of intervention, and a final assessment after 8 weeks of intervention [18]. Previous studies have shown that 6 weeks of balance exercise intervention and 2 months of body weight–bearing exercise can improve lower limb muscle strength, balance ability, and walking speed in older people [19,20]. We and other research groups thus consider 4-week and 8-week postintervention assessment time points as appropriate for this type of study.

We registered the trial with the University Hospital Medical Information Network Clinical Trials Registry (UMIN000053922; receipt number R000061550). The date of approval was March 22, 2024.

### Procedures

#### Recruitment

Participants will be recruited from among patients attending the collaborating hospitals. All 3 recruitment sites follow the same recruitment plan. Recruitment will continue until the target number of cases is achieved, with a 1-year time limit. At the end of a regular outpatient clinic session, a participant who meets all of the eligibility criteria and wishes to participate in the study will be provided with a description of the study, a questionnaire, and an evaluation sheet, and they will be asked to give their written informed consent to participate in the study. The need for follow-up and written consent for future contact will also be explained. The candidate participants are also informed that they can withdraw their consent or participation at any time if they so desire.

#### Intervention

**Figure 1:**
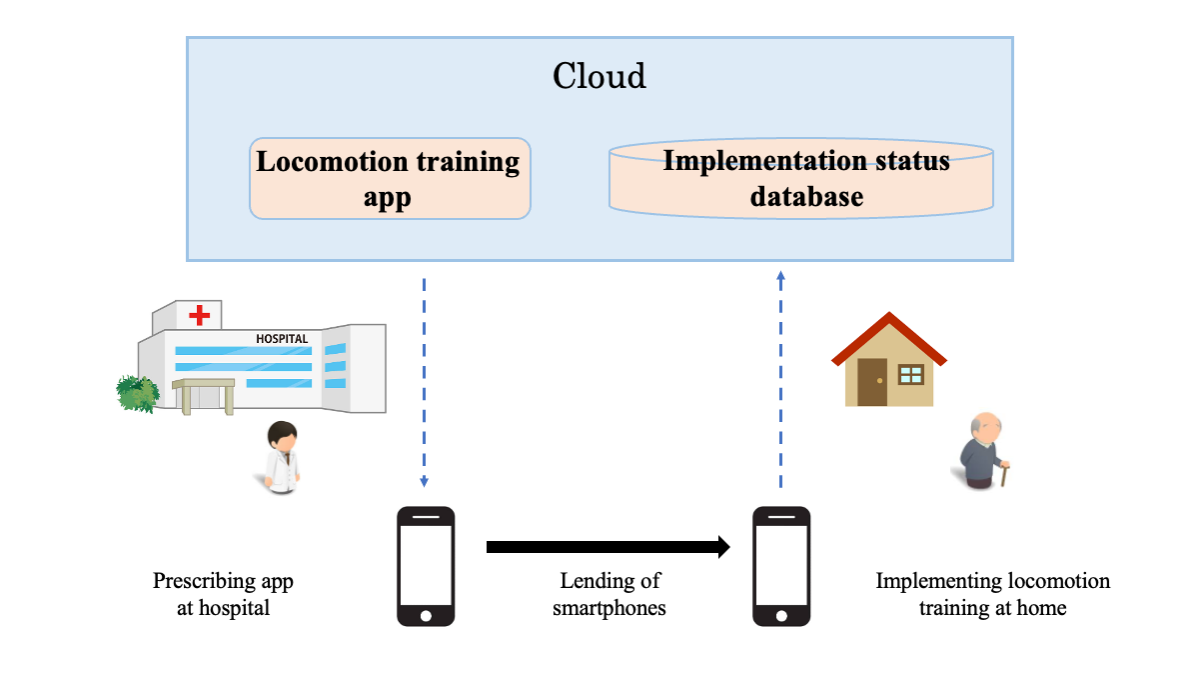
Implementation schema for the locomotive training (LT) app used in this study. The physician in charge will provide a smartphone onto which the LT app has been downloaded to the patient. The patients perform the LT at home, and the app’s implementation data are stored in the database and saved in the cloud. Through the cloud, the physician can check the participant’s LT implementation remotely.

The smartphone app used in this study is a newly prototyped app developed for this research study. We will provide smartphones equipped with a communication environment and downloaded apps in advance, which will be lent to the participants (Figure 1). The participants will be instructed to refrain from using the smartphone for any purpose other than use of the relevant smartphone app.

**Figure 2:**
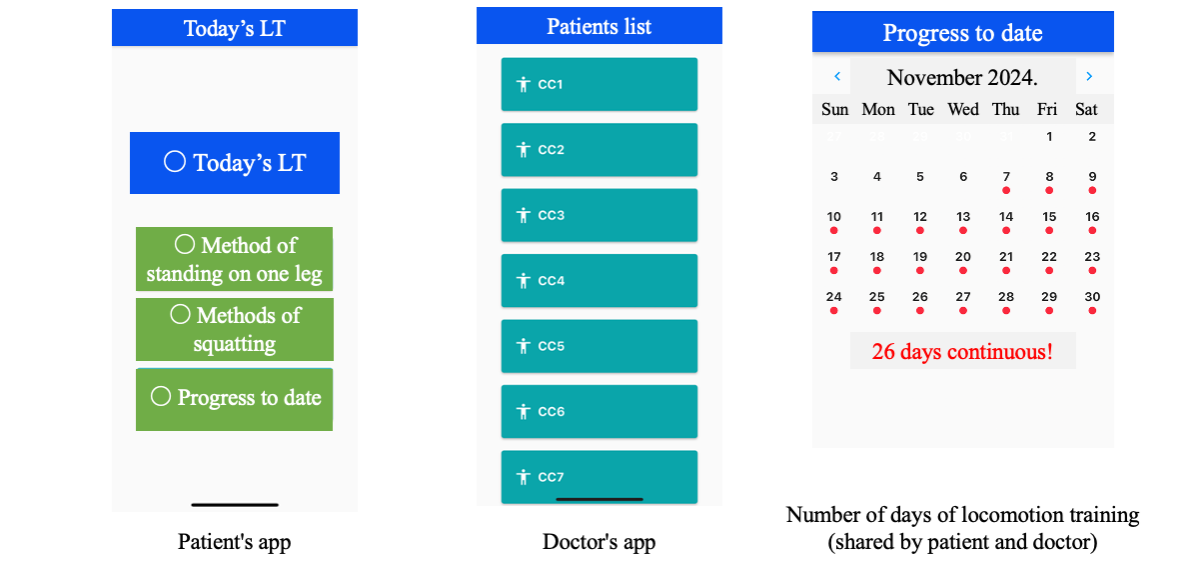
Screenshots of the app used in this study. The app for patients has a simple design to help them perform the locomotive training (LT). The app for physicians allows them to check a patient’s LT status based on the ID for that patient. The number of days with LT is displayed on the same screen for both the patient and the physician.

Each participant will be scheduled for LT intervention every day for 8 weeks. The LT application is simple to use and is set up so that by touching the screen, a voice announcement is played and the user can perform 2 LT programs: the 1-leg stand program and the squat program. A single LT session consists of a program of 60 seconds of standing on one leg and 5 squats, with each exercise repeated 3 times. For participants with weak legs and backs who find it difficult to do the regular LT, it is recommended that they stand on one leg while supporting their body with their hands and fingers on a desk, or that they sit on a chair and stand up by putting their hands on a desk instead of doing squats. In addition, for participants with particularly weak legs and backs, each LT repetition could also be reduced to 1 time instead of 3 times [5]. Sustainable goals are set for each individual for LT continuity. After completion of the LT, the participant touches the screen to check the record of the LT, not only on the participant’s but also on his or her physician’s smartphone (Figure 2).

### Outcomes

#### Timeline

The timetable of enrollment, interventions, and assessments is displayed in Table 1, including the quantitative self-reported outcomes and TUG results at the baseline, interim, and final evaluations. Demographic and clinical information will be obtained from the participants’ electronic medical records at baseline and will include their date of birth, gender, BMI, medical history, and disease or diseases causing LS.

**Table 1:** Timetable and evaluation items.

Item	Baseline	4 weeks after DTx^a^ initiation	8 weeks after DTx initiation
Patient consent	✓		
Patient background	✓		
Timed up and go test	✓	✓	✓
25-question Geriatric Locomotive Function Scale	✓	✓	✓
Behavioural Regulation in Exercise Questionnaire 3	✓	✓	✓
Days with locomotion training		✓	✓

^a^DTx: digital therapeutics.

#### Primary Outcome

The primary outcome is TUG test values before and after the DTx intervention. The TUG test is widely used in clinical practice as an outcome measure to assess mobility, fall risk, and dynamic balance in adults, and it can assess objective mobility [21,22]. Furthermore, previous research has shown that patients with LS have significantly longer TUG times than control groups [23]. In patients with rheumatoid arthritis, TUG values are significantly shortened and physical function is improved by continuing LT [24]. We determined that TUG is a useful indicator of the effects of LT for participants with LS.

#### Secondary Outcomes

Secondary outcomes are the participants’ GFLS-25 and BREQ-3 scores. The GFLS-25 is a self-report questionnaire used to assess difficulties and impairments in daily activities related to LS and can be used to assess subjective mobility [5-7]. The BREQ-3 assesses motivation for exercise with 18 questions in 6 areas: intrinsic motivation, integrative adjustment, identification adjustment, incorporative adjustment, external adjustment, and demotivation [25]. The BREQ-3 is used in this study to assess changes in attitudes toward exercise before and after the DTx intervention.

#### Other Clinical Outcomes

We will evaluate the numbers of days LT was performed at 4 weeks and 8 weeks after the intervention, as recorded in the smartphone app.

### Statistical Methods and Sample Size

The primary outcome of this study, TUG test values, will be measured repeatedly in the same participants at 0 weeks before the DTx intervention and 4 and 8 weeks after the intervention. These data will be compared with an ANOVA, with the TUG score as the dependent variable. The secondary outcomes, GLFS-25 and BREQ-3 scores obtained at the 3 time points, will also be compared with an ANOVA. The significance level will be set at .05 / 3 = .0167 with Bonferroni correction.

The minimal clinically important difference in the TUG test for patients with degenerative disc disease has been reported to be 3.4 (SD 5.0) [26]. Considering this finding, we set the minimal clinically important difference at 3 for this investigation. The required sample size was calculated using G*power (Heinrich-Heine-Universität Düsseldorf) [27]. We selected the corresponding *t* test, and we calculated the effect size as 0.6 based on a mean difference of 3.0 and an SD of difference of 5.0. Assuming that α is .05 and power (1 − β) is .80, the required sample size was estimated to be 24. We set the target number of cases for this study at 50, taking into account that the SD might be smaller than in previous studies, some participants might drop out, and other factors.

### Randomization and Blinding

This is a nonrandomized (unblinded) study and will use a single group. All participants will serve as their own controls; data from all participants before the DTx intervention will be used and compared with the data from after the DTx intervention. The planned statistical methods will add strength to the study’s conclusions.

### Data Monitoring and Adverse Event Reporting

The risk of adverse events from participation in DTx is very low. We are thus not establishing a data monitoring committee, and no interim analyses are planned to identify risks.

The principal investigator will (1) report the progress of the study without delay to the ethics review committee on an annual basis, (2) monitor adverse events related to the procedures and group program, and (3) discuss any necessary protocol amendments. If a member of the research team becomes aware of the occurrence of a serious adverse event, they will promptly report it to the research director, who will promptly report it to the principal investigator. If the principal investigator determines that the serious adverse event was unanticipated and is unable to be ruled out as being causally related to this study, he will promptly report it to the head of the research institution.

### Ethical Considerations

The Fukushima Medical University Ethics Review Committee provided ethical approval for this study (REC 2023-196). All study methods follow the relevant guidelines and regulations, and written informed consent will be obtained from participants. The study’s main results will be published in a peer-reviewed scientific journal and submitted for presentation at clinical and scientific meetings and conferences. The principal investigator will oversee the publication and presentation of the study results, including the determination of authorship. Participants will be asked if they would like to be informed about the study’s main results, and those who express interest and provide contact information will be provided this information as soon as it becomes available. Data will be deidentified and participant information will be protected. Although no monetary rewards or other compensation will be provided to those participating in the research, in the event of an adverse event, we will respond promptly and provide compensation while consulting with the ethics committee.

Participants’ data will be entered into a password-protected electronic file that will be kept on a secure computer by the principal investigator. Coding sheets with identifiable participant information will be stored on a secure computer as password-protected electronic files. Hard copies of consent forms and research documents will be stored in a locked filing cabinet at the research site.

## Results

Trial enrollment began in April 2024 and will initially continue until at least April 2025. As of April 2025, 47 had participants agreed to be evaluated, 47 participants had completed the interim evaluation, and there were complete data sets from 45 participants for the proposed analysis. The data analysis has not yet been conducted.

## Discussion

To our knowledge, this is the first trial to evaluate the effectiveness of DTx in preventing and ameliorating LS and changing the exercise behavior of older people. Although it is limited by its single-group design, the study has the strength that it evaluates the effectiveness of DTx in routine practice. External validity is also strengthened by the that fact that the practice sites are spread across 3 general hospitals and target communities with different sociodemographic characteristics.

A randomized design was not feasible, as the intervention is offered in routine practice [28], thus limiting causal conclusions. However, a randomized control study of approximately 200 participants per group in collaboration with local authorities has been proposed to strengthen this study. This is intended to improve the quality of the study by assessing in greater detail the impact of DTx on behavioral change and the prevention and amelioration of LS in older adults.

We are concerned that participants with low exercise awareness and low technology literacy may have difficulty participating in this study. However, for patients who are highly conscious of exercise but find it difficult to continue exercising on their own, or who are anxious about the content of the exercise, an advantage of the study is that they can exercise according to a set protocol with the cooperation of health care providers.

Notably, DTx enables the online assessment of the participants’ engagement in LT and facilitates feedback in routine practice. DTx is less burdensome for medical practitioners and more motivating for the participants as it creates an environment in which the participant is continuously supported by a physician. This research promises not only to lead to more efficient use of health care resources and reduced human and financial burdens, it may also lay the groundwork for more rapid implementation of DTx in real-world clinical practice, depending on the results. Nevertheless, a randomized controlled study based on our results will be required in order for DTx to gain insurance coverage in Japan, which calls for adequate funding and the cooperation of local authorities. If successful, the study will support the prevention and amelioration of LS and the establishment of better exercise habits among older adults.

## Abbreviations

BREQ-3: Behavioural Regulation in Exercise Questionnaire 3
DTx: digital therapeutics
GLFS-25: the 25-question Geriatric Locomotive Function Scale
JOA: Japanese Orthopaedic Association
LS: locomotive syndrome
LSC: locomotive syndrome check
LT: locomotion training
mHealth: mobile health
TUG: timed up and go

## Data Availability

The datasets generated during and analyzed in this study will be made available from the corresponding author on reasonable request.

## References

[1] Nakamura K (2009). Locomotive syndrome: disability-free life expectancy and locomotive organ health in a "super-aged" society. J Orthop Sci.

[2] Nakamura K (2008). A "super-aged" society and the "locomotive syndrome". J Orthop Sci.

[3] Ishibashi H (2018). Locomotive syndrome in Japan. Osteoporos Sarcopenia.

[4] Nakamura K, Ogata T (2016). Locomotive syndrome: definition and management. Clin Rev Bone Miner Metab.

[5] Yoshimura N, Muraki S, Oka H, Tanaka S, Ogata T, Kawaguchi H, Akune T, Nakamura K (2015). Association between new indices in the locomotive syndrome risk test and decline in mobility: third survey of the ROAD study. J Orthop Sci.

[6] Ogata T, Muranaga S, Ishibashi H, Ohe T, Izumida R, Yoshimura N, Iwaya T, Nakamura K (2015). Development of a screening program to assess motor function in the adult population: a cross-sectional observational study. J Orthop Sci.

[7] Taniguchi M, Ikezoe T, Tsuboyama T, Tabara Y, Matsuda F, Ichihashi N, Nagahama Study group (2021). Prevalence and physical characteristics of locomotive syndrome stages as classified by the new criteria 2020 in older Japanese people: results from the Nagahama study. BMC Geriatr.

[8] Muramatsu N, Akiyama H (2011). Japan: super-aging society preparing for the future. Gerontologist.

[9] Free C, Phillips G, Felix L, Galli L, Patel V, Edwards P (2010). The effectiveness of M-health technologies for improving health and health services: a systematic review protocol. BMC Res Notes.

[10] Patel NA, Butte AJ (2020). Characteristics and challenges of the clinical pipeline of digital therapeutics. npj Digit. Med.

[11] Kario K, Nomura A, Harada N, Okura A, Nakagawa K, Tanigawa T, Hida E (2021). Efficacy of a digital therapeutics system in the management of essential hypertension: the HERB-DH1 pivotal trial. Eur Heart J.

[12] Nomura A, Tateno H, Masaki K, Muto T, Suzuki S, Satake K, Hida E, Fukunaga K (2019). A novel smoking cessation smartphone app integrated with a mobile carbon monoxide checker for smoking cessation treatment: protocol for a randomized controlled trial. JMIR Res Protoc.

[13] Ramakrishnan P, Yan K, Balijepalli C, Druyts E (2021). Changing face of healthcare: digital therapeutics in the management of diabetes. Curr Med Res Opin.

[14] Akahane M, Maeyashiki A, Yoshihara S, Tanaka Y, Imamura T (2016). Relationship between difficulties in daily activities and falling: loco-check as a self-assessment of fall risk. Interact J Med Res.

[15] Keller K, Engelhardt M (2013). Strength and muscle mass loss with aging process. Age and strength loss. Muscles Ligaments Tendons J.

[16] Borson S, Scanlan J, Brush M, Vitaliano P, Dokmak A (2000). The mini-cog: a cognitive 'vital signs' measure for dementia screening in multi-lingual elderly. Int J Geriatr Psychiatry.

[17] Borson S, Scanlan JM, Chen P, Ganguli M (2003). The Mini-Cog as a screen for dementia: validation in a population-based sample. J Am Geriatr Soc.

[18] Imai F, Momino K, Katsuki F, Horikoshi M, Furukawa T, Kondo N, Toyama T, Yamaguchi T, Akechi T (2019). Smartphone problem-solving therapy to reduce fear of cancer recurrence among breast cancer survivors: an open single-arm pilot study. Jpn J Clin Oncol.

[19] Hirase T, Inokuchi S, Nakahara K, Matsusaka N (2011). Time course changes in physical function following different exercise interventions for community-dwelling elderly balance and muscle strength exercises. Rigakuryoho Kagaku.

[20] Krebs D, Scarborough D, McGibbon Chris A (2007). Functional vs. strength training in disabled elderly outpatients. Am J Phys Med Rehabil.

[21] Podsiadlo D, Richardson S (1991). J Am Geriatr Soc.

[22] Jeong S, Shin DW, Han K, Jung JH, Chun S, Jung H, Son KY (2019). Timed up-and-go test is a useful predictor of fracture incidence. Bone.

[23] Nishimura T, Imai A, Fujimoto M, Kurihara T, Kagawa K, Nagata T, Sanada K (2020). Adverse effects of the coexistence of locomotive syndrome and sarcopenia on the walking ability and performance of activities of daily living in Japanese elderly females: a cross-sectional study. J Phys Ther Sci.

[24] Mochizuki T, Yano K, Ikari K, Okazaki K (2023). Effectiveness of locomotion training in patients with rheumatoid arthritis: a prospective clinical trial. J Phys Ther Sci.

[25] Cid L, Monteiro D, Teixeira D, Teques P, Alves S, Moutão João, Silva M, Palmeira A (2018). The Behavioral Regulation in Exercise Questionnaire (BREQ-3) Portuguese-Version: evidence of reliability, validity and invariance across gender. Front Psychol.

[26] Gautschi OP, Stienen MN, Corniola MV, Joswig H, Schaller K, Hildebrandt G, Smoll NR (2017). Assessment of the minimum clinically important difference in the timed up and go test after surgery for lumbar. Neurosurgery.

[27] Faul F, Erdfelder E, Lang A, Buchner A (2007). G*Power 3: a flexible statistical power analysis program for the social, behavioral, and biomedical sciences. Behav Res Methods.

[28] Craig P, Dieppe P, Macintyre S, Michie S, Nazareth I, Petticrew M, Medical Research Council Guidance (2008). Developing and evaluating complex interventions: the new Medical Research Council guidance. BMJ.

